# Association between Socioeconomic Position and Tuberculosis in a Large Population-Based Study in Rural Malawi

**DOI:** 10.1371/journal.pone.0077740

**Published:** 2013-10-21

**Authors:** Anna Odone, Amelia C. Crampin, Venance Mwinuka, Simon Malema, J. Nimrod Mwaungulu, Lumbani Munthali, Judith R. Glynn

**Affiliations:** 1 London School of Hygiene and Tropical Medicine, London, United Kingdom; 2 University of Parma, School of Medicine, Parma, Italy; 3 Karonga Prevention Study, Chilumba, Malawi; 4 Department of Global Health and Social Medicine, Harvard Medical School, Boston, Massachusetts, United States of America; Institute of Infectious Diseases and Molecular Medicine, South Africa

## Abstract

**Setting:**

There is increasing interest in social structural interventions for tuberculosis. The association between poverty and tuberculosis is well established in many settings, but less clear in rural Africa. In Karonga District, Malawi, we found an association between *higher* socioeconomic status and tuberculosis from 1986-1996, independent of HIV status and other factors.

**Objective:**

To investigate the relationship in the same area in 1997-2010.

**Design:**

All adults in the district with new laboratory-confirmed tuberculosis were included. They were compared with community controls, selected concurrently and frequency-matched for age, sex and area.

**Results:**

1707 cases and 2678 controls were interviewed (response rates >95%). The odds of TB were increased in those working in the cash compared to subsistence economy (p<0.001), and with better housing (*p-*trend=0.006), but decreased with increased asset ownership (*p-*trend=0.003). The associations with occupation and housing were partly mediated by HIV status, but remained significant.

**Conclusion:**

Different socioeconomic measures capture different pathways of the association between socioeconomic status and tuberculosis. Subsistence farmers may be relatively unexposed whereas those in the cash economy travel more, and may be more likely to come forward for diagnosis. In this setting “better houses” may be less well ventilated and residents may spend more time indoors.

## Introduction

The scientific and public health communities have recently given increasing attention to the social determinants of tuberculosis (TB) [1,2]. There is general agreement that action on the social determinants of TB should be developed around three main axes: i) health sector interventions, ii) intersectoral policies, and iii) research aimed at identifying and measuring their association with TB [3].

While the association between socioeconomic position (SEP) and TB in high-income countries is well documented [4-9], few studies have investigated the socioeconomic risk factors associated with TB in low-income settings [10-16]. A study conducted in Karonga District, Northern Malawi in 1986-1996 showed an unexpected association between TB and measures of higher SEP [13]. This positive association persisted after adjusting for age, sex and HIV. In Zambia, tuberculosis infection was associated with higher SEP [10], but tuberculosis disease, as detected in a prevalence survey, was associated with lower household SEP, partly mediated through food insecurity [11]. 

The relationship between deprivation and TB might be different in rural Africa as compared to high-income settings. It will reflect a combination of opportunities for infection (increased by travel, crowding, poor ventilation); susceptibility to disease (increased by HIV and malnutrition); and likelihood of diagnosis (increased by education and proximity to clinics) [14,17,18]. Assessing the direction and the mediating factors of such an association is of fundamental importance for guiding effective preventive measures and control programmes.

We investigated the relationship between socioeconomic factors and TB in Karonga District, Malawi for the period 1997-2010.

## Methods

Ethics statement: ethics approval for the study was received from the Health Sciences Research Committee, Malawi, and the ethics committee of the London School of Hygiene & Tropical Medicine, UK. Individual written consent was sought from cases and controls, with separate written consent for HIV testing. 

A series of large-scale population-based case-control studies were conducted as part of The Karonga Prevention Study in northern Malawi to investigate the changing role of HIV, the importance of household contact and other risk factors for TB [19-22]. Here we assess associations with socio-economic factors. The source population underlying the current study is the general population aged ≥15 years of the whole Karonga District from 1997 to 2010.

Subjects were included in the study as cases if they had a diagnosis of confirmed or probable TB, and had not had TB previously, and were resident in the district [23]. Pulmonary TB was defined as confirmed or probable if they had at least one positive smear or culture (excluding those with only a single smear with <10 acid fast bacilli/100 fields). Extra-pulmonary TB was defined as confirmed or probable if there was a positive result from smear, culture or biopsy [23]. 

All incident TB cases diagnosed in the district during the study period were included in the study. Case ascertainment was carried out through a system of ‘enhanced’ passive TB surveillance [23].

Controls were concurrently selected from 1998 onwards. They were frequency matched to cases on sex, age, population density and area. A field-based random sampling method, described in detail elsewhere [24], was used to select controls from the source population. This used random starting places in the district, weighted by population density, and a spinning top to choose a random direction for the field teams to walk to find controls of the pre-specified sex and age band.

Exposures were assessed through questionnaires administered in-person after informed consent was given. Individual-level variables, including education level, and occupation, were collected for the entire study period. Cases were interviewed in hospital or health facility and asked about exposures before the onset of symptoms [23]. Controls were interviewed at home. Household-level variables were collected during home visits to both cases and controls for the period 1998-2005. A dwelling score and an asset index were built to classify households in different socioeconomic categories as described elsewhere [25]. The dwelling score depended on house construction (eg high scores for cement floors and tin (“iron”) or tile roofs, low for mud floors and thatch). The asset score was based on the average monetary value of a number of commonly owned assets. Occupation of the head of household was also considered as a measure of household SEP. HIV testing of cases and controls was carried out after counseling and if consent was given [23]. Results were reported to the individuals unless they did not want to know them.

**Figure 1:**
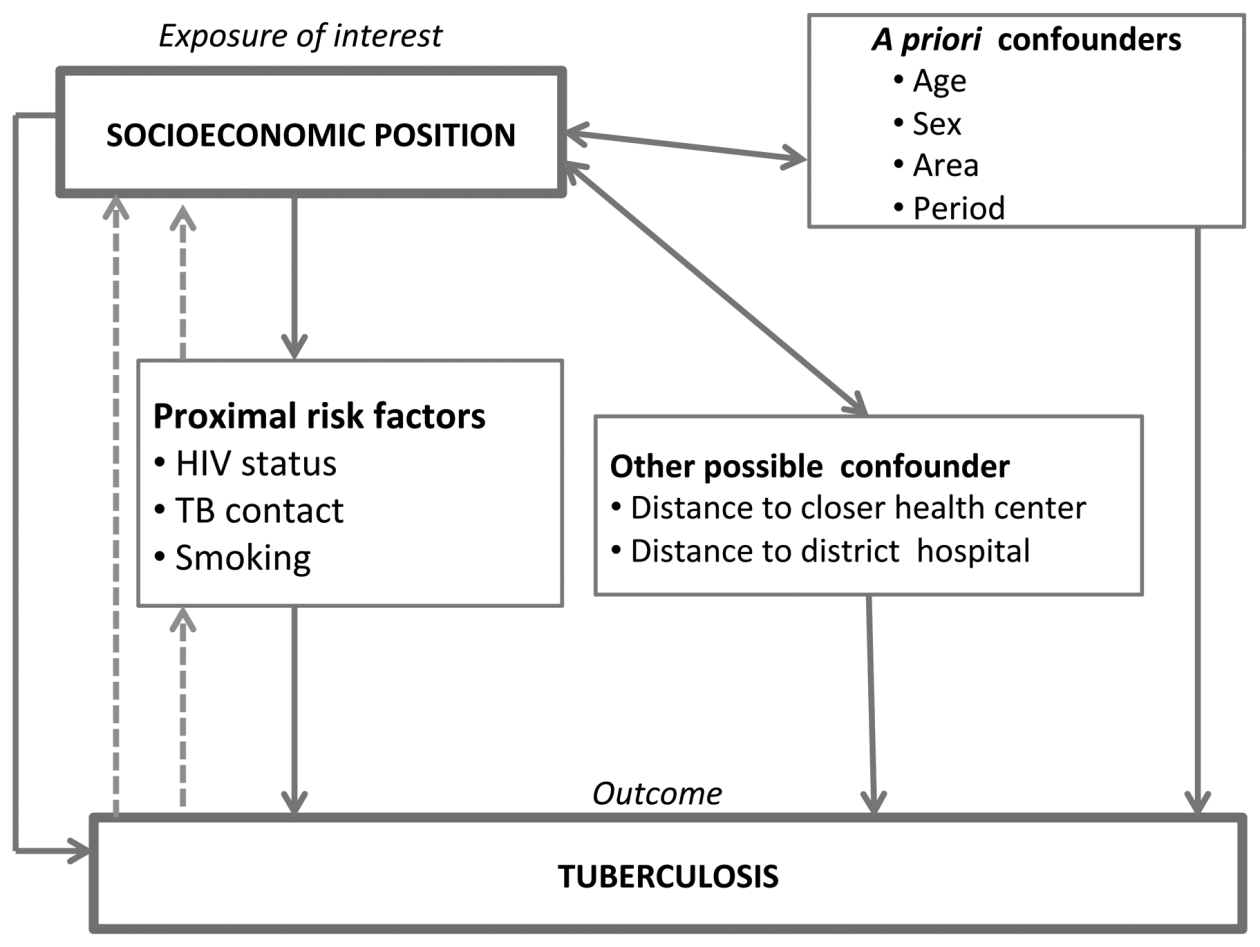
Conceptual framework. The conceptual framework describes the association between socioeconomic position (SEP) and tuberculosis (TB) disease in terms of distal and proximal risk factors, *a* *priori* confounders and other possible confounders. * dotted line = “reverse causality”, not explored as not relevant for incident cases (see *Discussion* section).

On the basis of the literature on biological and social determinants of TB, a conceptual framework [26] was developed to describe the association between SEP and TB disease in terms of distal and proximal risk factors, a priori confounders and other possible confounders (Figure 1). The main exposures of interest were SEP variables. SEP was considered a distal determinant of TB [26], and it was hypothesized that some of the effect of SEP could be mediated through behavioural (smoking) [27], biological (HIV) [27,28] and transmission (TB contact) [27] risk factors. The modelling strategy was built on the basis of the conceptual framework. The whole dataset was used to explore the association between TB and SEP at the individual level. The 1998-2005 dataset was used to explore the association between TB and SEP at the household level. Effect estimates were expressed as unadjusted and adjusted odds ratios (ORs) with their 95% confidence intervals (95%CI) and were derived from univariable and multivariable logistic regression modelling. When exploring the mediation pathway, a reduction in the SEP effect estimate after inclusion of proximal risk factors in the models was considered as evidence of mediation [10,11]. HIV status was not available for 12% of cases and 18% of controls. As this is such an important risk factor for TB, and in order to fully account for the effect of HIV, the regression analyses exploring mediation of SEP by HIV were restricted to subjects with known HIV status as well as to subjects with non-missing SEP variables. 

## Results

Between 1997 and 2010, 1,707 TB cases were identified in Karonga. A total of 2,678 controls were included. All cases and 96% of selected controls were interviewed. Demographic characteristics were available for all study subjects. Among cases, 50.8% were females. The mean age of cases was 37 years (SD=13). Because of the frequency-matched design and concurrent selection of controls, age, sex and calendar period distribution were similar for cases and controls. 

The socioeconomic profile of the study population at the individual level is presented in Table 1. Fewer than 10% of both cases and controls had no education or had attended secondary/tertiary-level education. The majority of study participants were subsistence farmers. In the univariable analysis, the odds of TB increased with increasing levels of education (test for trend, p<0.001). Being employed in small businesses/trade/manual work and being salaried/employed in large businesses were associated with increasing odds of TB, ORs being 2.04 (95%CI:1.67-2.50) and 2.19 (95%CI:1.75-2.75), respectively, compared to farmers.

**Table 1:** Individual-level risk factors for Tuberculosis, Karonga district: 1997-2010.

	**CASES (N=1707)**	**CONTROLS (N=2678)**	**OR (95%CI)-Unadjusted**		**OR (95%CI**)**- Adjusted for *a priori* confounders** ^$^	
	no.(%)	no.(%)				
**Schooling**						
None	102(5.98)	218(8.14)	1.00	p=0.003	1.00	p=0.04
1-5 years primary	592(34.68)	1094(40.85)	1.16(0.90-1.49)		0.88 (0.67-1.16)	
6-8 years primary	703(41.18)	1183(44.17)	1.27(0.99-1.64)		0.98 (0.74-1.29)	
Secondary/tertiary	155(9.08)	179(6.68)	1.85(1.34–2.55)		1.29 (0.90-1.84)	
Missing	155(9.08)	4(0.15)				
**Individual occupation**						
Farmer/fisherman	897(52.55)	1793(66.95)	1.00	p<0.001	1.00	p<0.001
Not working/child/retired/casual	207(12.13)	476(11.77)	0.86(0.72-1.04)		0.83 (0.68-1.01)	
Manual/trade/small business	226(13.24)	221(8.25)	2.04(1.67-2.50)		1.95 (1.57-2.43)	
Salaried/large business	181(10.60)	165(6.16)	2.19(1.75-2.75)		2.16 (1.69-2.76)	
Missing	196(11.48)	23(0.86)				
**HIV status**						
HIV-	619(36.26)	1854(69.23)	1.00	p<0.001	1.00	p<0.001
HIV+	883(51.73)	335(12.51)	7.89(6.63-9.40)		9.28 (7.8-11.03)	
Missing	205(12.01)	489(18.26)				
**Smoking habit (ever smoked)**						
No	1120(65.61)	2155(80.47)	1.00	p<0.001	1.00	p=0.15
Yes	170(9.96)	473(17.66)	0.69(0.57-0.84)		0.85 (0.68-1.06)	
Missing	417(24.43)	50(1.87)				
**TB contact**						
None	967(56.65)	1864(69.60)	1.00	p<0.001	1.00	p<0.001
Yes, outside the family	173(10.13)	343(12.81)	0.97(0.80-1.19)		0.82 (0.66-1.02)	
Yes, within the family	441(25.83)	369(13.78)	2.3(2.00-2.80)		2.31 (1.96-2.73)	
Both	75(4.39)	95(3.55)	1.52(1.11-2.01)		1.25 (0.90-1.74)	
Missing	51(2.99)	7(0.26)				
**Distance to closest health centre**						
<2(Km)	462(27.07)	699(26.10)	1.00	p=0.6	1.00	p=1.0
2-4	813(47.63)	1263(47.16)	0.97(0.84-1.13)		0.99 (0.84-1.15)	
>4	430(25.19)	706(26.36)	0.92(0.78- 1.09)		0.98 (0.82-1.18)	
Missing	2(0.12)	10(0.37)				
**Distance to district hospital**						
<2(Km)	249(14,59)	354(13.22)	1.00	p=0.003^a^	1.00	p=0.3
-10	538(31.52)	730(27.26)	1.05(0.86-1.28)		1.1 (0.89-1.36)	
10-25	458(26.83)	820(30.62)	0.79(0.65-0.97)		0.89 (0.68-1.17)	
>25	460(26.95)	760(28.38)	0.86(0.70-1.05)		0.97 (0.74-1.25)	
Missing	2(0.12)	14(0.52)				

OR = odds ratio, CI = confidence intervals

$ = adjusted for age, sex, area and calendar period.

All p-values obtained through Likelihood ratio test (LRT)

More than half the cases were HIV positive compared to 12.5% of the controls, giving an OR of 7.89 (95%CI:6.63-9.40). TB contact within the family was associated with 2.3 times increase in the odds of TB. 

After adjusting for *a priori* confounders (age, sex, area and calendar period), there was still strong evidence of association between individual occupation and TB while there was weak evidence of association between education and TB. The multivariable analysis of the association between SEP and TB at the individual level is presented in Table 2. 

**Table 2:** Individual-level socioeconomic risk factors for Tuberculosis.

	**CASES** ^*^ **(N=1344**)	**CONTROLS** ^*^ **(N=2169**)	**OR^1^ (95%CI)**		**OR** ^2^ (**95%CI**) **Exploring HIV mediation**	
	no.(%)	no.(%)				
**Schooling**						
None	95(7.07)	158(7.28)	1.00	p=0.4	1.00	p=0.3
1-5 years primary	522(38.84)	877(40.43)	0.77(0.57-1.04)		0.74(0.53-1.03)	
6-8 years primary	615(45.76)	994(45.83)	0.82(0.61-1.11)		0.73(0.52-1.02)	
Secondary/tertiary	112(8.33)	140(6.45)	0.81(0.54-1.23)		0.69(0.43-1.09)	
**Individual occupation**						
Farmer/fisherman	806(59.97)	1438(66.30)	1.00	p<0.001	1.00	p=0.04
Not working/child/retired/casual	186(13.84)	403(18.58)	0.79(0.64-0.98)		0.89(0.7-1.13)	
Manual/trade/small business	207(15.40)	191(8.81)	1.90(1.50-2.41)		1.44(1.11-1.88)	
Salaried/large business	145(10.79)	137(6.32)	1.94(1.46-2.59)		1.46(1.06-2.01)	

Exploring the mediation effect of HIV. Karonga district: 1997-2010.

OR = odds ratioCI = confidence intervals

* subset of subjects with known HIV status

1 adjusted for age, sex, area, calendar period, distance to closest health centre, distance to district hospital

2 adjusted for age, sex, area, calendar period, distance to closest health centre, distance to district hospital and HIV

Note: all p-values obtained through Likelihood ratio test (LRT)

There was still evidence of a positive association between TB and being employed in small businesses/trade/manual work (OR=1.90, 95%CI:1.50-2.41) and being salaried/employed in large businesses (OR=1.94, 95%CI:1.46-2.59) compared to subsistence farming. Adjusting for distance to closest heath centre and the hospital made little difference to the results. HIV partly mediated the associations with these jobs in the cash economy although, after including HIV in the model, there was still evidence of an association (Table 2). Neither of the other hypothesized mediating factors (smoking and TB contact) led to changes in the association between TB and education or occupation (not shown).

The 1998-2005 subset used to analyse the association between TB and household-level SEP included 867 cases and 1,788 controls. The distribution of socio-demographic characteristics, HIV, smoking habit and distance to health centres was similar to that reported for the whole dataset. At the univariable level, increasing dwelling scores were associated with increased odds of TB (test for trend, p=0.002) (Table 3). A weak trend in the opposite direction was evident between asset index and TB. With regard to occupation of head of household, being salaried or employed in large business was associated with increased odds of TB (OR=1.25, 95%CI:1.01-1.56). After adjusting for *a priori* confounders (age, sex, area and calendar period), effect estimates for housing, asset possession and occupation of head of household were only minimally changed (Table 3).

**Table 3:** Household-level socioeconomic risk factors for Tuberculosis, Karonga district: 1998-2005.

	**CASES (N=867)**	**CONTROLS (N=1788)**	**OR (95%CI)-Unadjusted**		**OR (95%CI**)**- Adjusted for *a priori* confounders** ^$^	
	no.(%)	no.(%)				
**Dwelling score**						
1(lowest)	174(20.7)	534(29.87)	1.00	p=0.002^a^	1.00	p=0.008^a^
2	316(36.45)	739(41.33)	1.31(1.06-1.63)		1.29 (1.02-1.62)	
3	163(18.80)	344(19.24)	1.45(1.13-1.87)		1.49 (1.13-1.96)	
4(highest)	72(8.30)	145(8.11)	1.52(1.10-2.12)		1.43 (1.00-2.03)	
Missing	142(16.38)	26(1.45)				
**Asset index**						
1(lowest)	149(17.19)	318(17.79)	1,00	p=0.11^a^	1.00	p=0.10^a^
2	119(13.73)	271(15.16)	0.94(0.7-1.25)		0.97 (0.72-1.31)	
3	146(16.84)	376(21.03)	0.83(0.63-1.09)		0.87 (0.66-1.16)	
4	183(21.11)	450(25.17)	0.87(0.67-1.3)		0.87 (0.67-1.14)	
5(highest)	133(15.34)	353(19.74)	0.80(0.61-1.06)		0.80 (0.60-1.07)	
Missing	137(15.80)	20(1.12)				
**Occupation of head of household**						
Farmer/fisherman	591(68.17)	1274(71.25)	1.00	p=0.04	1.00	p=0.04
Not working/ child/retired/casual	16(1.85)	54(3.02)	0.64(0.36-1.13)		0.54 (0.30-0.97)	
Manual/trade/small business	82(9.46)	207(1.58)	0.85(0.65-1.12)		0.78 (0.58-1.05)	
Salaried/large business	146(16.61)	249(13.93)	1.25(1.10-1.56)		1.09 (0.85-1.39)	
Missing	34(3.92)	4(0.22)				

OR = odds ratio, CI = confidence intervals

a test for trend

adjusted for age, sex, area and calendar period.

All p-values obtained through Likelihood ratio test (LRT)

The multivariable analysis of the association between SEP and TB at the household level is presented in Table 4. After adjusting for confounders, including individual-level SEP measures and distance to health centre and hospital, increasing dwelling scores were still associated with increased odds of TB, odds being respectively 29%, 36% and 56% higher in subjects with dwelling score 2, 3 and 4 as compared to the lowest value. In contrast, higher asset indices were associated with decreased odds of TB, odds being respectively 18%, 24% and 35% lower in subjects with asset index 3, 4 and 5 as compared to the lowest value. These patterns were maintained after adding smoking and TB contact in the regression models (not shown). HIV appeared to partly mediate the relationship between dwelling score and TB and adjusting for HIV strengthened the trend with asset score. Associations with occupation of head of household were weak after adjusting for HIV. 

**Table 4:** Household-level socioeconomic risk factors for Tuberculosis.

	**CASES** ^*^ **(N=567**)	**CONTROLS** ^*^ **(N=1,410**)	**OR^1^ (95%CI)**		**OR^2^ (95%CI)**	
	no.(%)	no.(%)			**Exploring HIV mediation**	
**Dwelling score**						
1(lowest)	143(25.22)	425(30.14)	1.00		1.00	
2	256(44.97)	587(41.63)	1.29(0.98-1.68)	p=0.04^a^	1.41(1.05-1.89)	p=0.2^a^
3	117(20.63)	281(19.93)	1.36(0.96-1.93)		1.20(0.82-1.76)	
4(highest)	52(9.17)	117(8.30)	1.56(0.96-2.52)		1.40(0.82-2.37)	
**Asset index**						
1(lowest)	115(20.28)	244(17.30)	1.00		1.00	
2	98(17.28)	207(14.68)	1.10(0.77-1.56)	p=0.003^a^	0.98(0.67-1.45)	p=0.002^a^
3	113(19.93)	306(21.70)	0.82(0.59-1.14)		0.76(0.52-1.09)	
4	138(24.34)	370(26.24)	0.76(0.54-1.05)		0.66(0.46-0.95)	
5(highest)	103(18.17)	283(20.07)	0.65(0.46-0.93)		0.61(0.41-0.90)	
**Occupation of head of household**						
Farmer/fisherman	417(73.54)	996(70.64)	1.00		1.00	
Not working/ child/retired/casual	10(1.76)	41(2.91)	0.58(0.27-1.25)	p=0.006	0.62(0.28-1.41)	p=0.2
Manual/trade/small business	51(8.99)	179(12.70)	0.50(0.32-0.78)		0.61(0.38-1.00)	
Salaried/large business	89(15.70)	194(13.76)	0.68(0.45-1.03)		0.84(0.53-1.34)	

Exploring the mediation effect of HIV. Karonga district: 1998-2005.

OR = odds ratioCI = confidence intervals

* subset of subjects with known HIV status

1 adjusted for age, sex, area, calendar period, distance to closest health centre, distance to district hospital, education, individual occupation.

2 adjusted for age, sex, area, calendar period, distance to closest health centre, distance to district hospital, education, individual occupation and HIV.

a test for trend

Note: all p-values obtained through Likelihood ratio test (LRT)

## Discussion

Our results show that TB was more common in those working in the cash economy than those in the subsistence economy. At the household level, TB was more common in those living in better built houses. In contrast, households with more assets had lower odds of TB. When exploring the mediation pathway, there was little evidence that either smoking habit or close contact with known TB cases explained any of the association between TB and the variables of interest. As expected, HIV was a major risk factor for TB in the area and acted as mediating proximal risk factor, partly but not completely explaining the association with occupation and house construction. 

The results of the current study are in line with previous findings from the same study setting [13]. One possible interpretation of our findings is that jobs other than subsistence farming or fishing might involve increased travelling (in crowded minibuses), socializing and working in indoor environments and thus be associated with increased risk of exposure to *M.tuberculosis* transmission. Although public sector health care in Malawi is free, there are travel and opportunity costs and these may be more easily afforded by those in the cash economy, leading to increased likelihood of diagnosis. In other African rural settings no association was shown between employment category and TB [15] while other studies provided some evidence that unemployment increased lifetime risk of TB [14]. 

Housing quality and asset index are both markers of household wealth, and were correlated with each other to some degree as would be expected (r^2^=12%, *p*<0.001, in a linear regression analysis, results not shown). However there was considerable variation: within each category of housing quality there were households with the full range of asset scores, and vice versa. Housing quality and asset index had opposite associations with TB. While household wealth would be expected to be related to lower TB risk, in our study setting, better housing quality may involve less ventilation (glass windows, more solid materials) and also, as another plausible pathway, residents of better houses may spend more time socializing indoors. In this climate there is no need to shelter from the cold and time spent indoors will increase with the likelihood that the house is well-lit and comfortable. This would increase the risk of *M.tuberculosis* transmission given the presence of an index case. A similar interpretation of the effect of housing quality was given by other authors both in similar [10] and different [29] settings. It is also possible that those with better houses may attract more dependents, and thus tend to be more overcrowded. The direction of the association between asset index and TB was in line with previous findings from other African settings and rural China [14,16]. Diabetes, which is a risk factor for TB, and is associated with relative affluence in poor settings, is likely to have been rare in the population at the time of the study [30-32], but it was not measured. 

The choice of appropriate SEP indicators in the context of TB studies has recently been debated in the literature [33]. Categorization of occupation in low-income settings where informal, seasonal and domestic work are more common than formal employment might pose problems and therefore ‘occupation’ as an exposure variable may fail to correctly assess social stratification [34]. The rationale for using setting-specific asset index and ad-hoc built dwelling score was to use exposure variables already used and validated in the same study setting [25], which were specific for the study population and relevant for the disease studied.

For the purpose of the study we developed a conceptual framework and used it to guide our analysis and interpret our results. This is considered a useful approach to explore causal inference, test pathway-specific hypotheses and plan and evaluate targeted public health interventions [35]. Few other studies have analysed the association between SEP and TB using a hierarchical conceptual framework [10,14,36,37].

An important limitation of our study is that we lacked information on proximal risk factors that have been shown to be important mediators in the relationship between SEP and TB in other settings, including malnutrition, food availability, alcohol consumption and co-morbidities [38]. Although TB disease can influence SEP of patients and their households [39,40], since we restricted the analysis to new TB cases with no previous diagnosis of TB, reverse causality is unlikely to be important in accounting for associations between TB and SEP. Although cases were interviewed in hospital, and controls at home, the assessment of household level indicators was done during household visits for both cases and controls.

## Conclusion

This study provides evidence that the risk of TB varies in different socioeconomic strata of the population in rural Malawi. In addition, it shows how different SEP measures capture different pathways of the association between SEP and TB and how HIV, more than other risk factors, partly mediates this association. 

In a historical moment when policy makers are willing to commit to address the social determinants of TB, it is of fundamental importance to gain solid epidemiologic evidence on the strength, direction and pathways of the association between SEP and TB. Studies like this could help to guide effective preventive measures as well as ‘TB-sensitive’ and ‘TB-specific’ social protection interventions [1].
